# Adaptation and validation of a scale of self‐efficacy and social support for physical activity in Spanish patients with severe mental disorders

**DOI:** 10.1002/brb3.1510

**Published:** 2019-12-26

**Authors:** David Perez‐Cruzado, Elisa Vera‐Garcia, Fermin Mayoral‐Cleries, Juan Vicente Luciano, Antonio Cuesta‐Vargas

**Affiliations:** ^1^ Department of Physiotherapy Grupo de Investigacion de Clinimetria IBIMA Primary Care Prevention and Health Promotion Research Network RedIAPP University of Malaga Malaga Spain; ^2^ Departmen of Occupational Therapy Catolic University of Murcia Murcia Spain; ^3^ Grupo de Investigación de Salud Mental IBIMA Hospital Regional de Málaga Malaga Spain; ^4^ Institut de Recerca Sant Joan de Déu Esplugues de Llobregat, Barcelona Spain; ^5^ Teaching, Research & Innovation Unit Parc Sanitari Sant Joan de Déu St. Boi de Llobregat Spain; ^6^ Primary Care Prevention and Health Promotion Research Network RedIAPP Madrid Spain; ^7^ School of Clinical Science Faculty of Health Science Queensland University Technology Brisbane Qld Australia

**Keywords:** physical activity, self‐efficacy, severe mental disorder, social support

## Abstract

**Background:**

People with severe mental disorders (SMDs) suffer problems of obesity, a sedentary life, and poor physical condition, mainly due to low levels of physical activity. Self‐efficacy (SE) and social support (SS) are important components that influence participation in physical activity.

**Methods:**

This study adapted a scale to assess SE and SS in promoting physical activity in Spanish people with SMDs, as well as provide preliminary evidence of its validity. One hundred Spanish patients (23% female) with SMDs, between 26 and 61 years old, completed the SE/SS assessment for SMD (SE/SS‐ASMD).

**Results:**

The instrument seemed to capture a four‐factor structure in people with SMDs. Due to the lack of a gold standard, the scale was related to other instruments with which it might be expected to show a correlation, such as those for physical activity and its quality; however, the levels of correlation found were low (≈0.3). The Internal consistency (Cronbach's α) for the SE‐ASMD, SS‐ASMD staff, SS‐ASMD peers, and SS‐ASMD family scales were 0.76, 0.76, 0.80, and 0.80, respectively.

**Conclusions:**

The psychometric analysis of the SE/SS‐ASMD supported its suitability as a new tool for researchers in the area of physical activity among people with SMDs.

## Graphical abstract

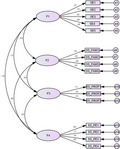

## 1 INTRODUCTION

The importance of physical activity for the physical fitness and psychological health of individuals with schizophrenia is well established (Carpiniello, Primavera, Pilu, Vaccargiu, & Pinna, 2013; Perez‐Cruzado, Cuesta‐Vargas, Vera‐Garcia, & Mayoral‐Cleries, 2018). Unfortunately, many people with schizophrenia remain inactive (Vancampfort et  al., 2016), and researchers and clinicians are challenged with how to help them initiate physical activity and maintain it long‐term. Previous research has found that one significant contributing factor to inactivity is social isolation (Soundy, Freeman, Stubbs, Probst, & Vancampfort, 2014).

Physical activity is associated with a large number of important health benefits, such as a decrease in the risk of diabetes, coronary heart disease, and death (Helmrich, Ragland, Leung, & Paffenbarger, 1991; Leon, Connett, Jacobs, & Rauramaa, 1987; Paffenbarger, Hyde, Wing, & Hsieh, 1986). Different studies have indicated that individuals with severe mental disorders (SMDs) carry out insufficient physical activity to benefit their health (Shor & Shalev, 2014; Vancampfort, Stubbs, Venigalla, & Probst, 2015); consequently, this population has high rates of obesity and coronary heart disease (Naslund et al., 2015; Osborn, Nazareth, & King, 2007).

Given this situation, there is a need to develop interventions to promote and foster physical activity in this population. Social cognitive theory (SCT) developed by Bandura has influenced a large amount of research on health behavior and the promotion of physical activity (Ariyabuddhiphongs & Chanchalermporn, 2007; Gao, 2012). Specifically, self‐efficacy (SE) and social support (SS) are two important elements of SCT in research of physical activity.

Self‐efficacy refers to individual action, in which a person believes in the possibility of achieving a certain desired result, and they can actively direct the course of their lives. The perception of SE is a very important predictor of healthy behavior, as well as self‐management of chronic disease (Clark et  al., 2015; Willis, 2015). When applied to the practice of physical activity, SE is usually considered to be the optimistic self‐belief of being able to overcome perceived challenges. SE has been related to the intention of people to practice physical activity, to strategize in addressing a sedentary lifestyle, and to maintain a regular practice of physical activity (Barz et al., 2015; Bergström, Börjesson, & Schmidt, 2015; Huffman, Pieper, Hall, St Clair, & Kraus, 2015; Plotnikoff, Gebel, & Lubans, 2014). Studies have also indicated that SE correlates with physical activity in adults with SMDs (Bezyak, Berven, & Chan, 2011).

Another important variable in terms of physical activity from the perspective of SCT is SS. Studies have indicated that SS in the general population critically influences the participation of individuals in physical activity (Draper, Grobler, Micklesfield, & Norris, 2015; Mendonça & Farias Júnior, 2015; Zhang et al., 2015). Other studies have also indicated the importance of social interaction in promoting physical activity in those with SMDs (Gray et al., 2014; Hoffmann et al., 2015). SS can come from several different groups, such as family and friends, in various forms, for example, emotional and financial help. Previous reports indicate that the SS received by people with SMDs that contributes to increased exercise comes mainly from three groups, family, professionals, and peers (Citrome & Yeomans, 2005; Daumit et al., 2005).

We studied the relationship between interventions and physical activity in a population that traditionally shows low levels of activity, those with intellectual disabilities. We made use of a previously developed SE and SS for activity scale to measure the effect of these variables on the performance of physical activity (Lee, Peterson, & Dixon, 2010).

A Spanish version of the SE and SS assessment for SMDs (SE/SS‐ASMD) should have immense applicability, since Spanish is the third most spoken language ​​in the world. In Spain, 9% of the general population (>400,000 people) suffer from a SMD (Muñoz, Perez‐Santos, Crespo, & Guillen, 2009). Therefore, the objective set out in the present study was the adaptation and validation of the SE/SS‐ASMD questionnaire for the promotion of physical activity in people with SMDs.

## 2 METHODS

### 2.1 Participants

The participants were recruited over a six‐month period from the mental health service of the Regional University Hospital of Málaga, Spain. A total of 100 adults with a SMD participated in this study and had a set of pathologies with an ICD‐10 diagnosis of an affective or nonaffective functional psychotic disorder (codes F20‐F22, F24, F25, F28‐F31, F32.3, and F33.3). The sample was 23% female and had a mean age of 45.39 years (±8.23; range 26–61 years). The inclusion criteria were individuals who: (a) were aged between 18 and 65 years; (b) had not suffered a psychotic crisis in the last four weeks; and (c) did not have any cardiovascular, neuromuscular, or endocrine pathologies that prevented them from performing physical exercise or that limited their physical fitness. The characteristics of participants are described in Table 1.

**Table 1:** Characteristics of participants

	Mean	*SD*
Age	46.21	8.37
BMI	29.13	5.81
Height (cm)	164.77	20.81
Weight (kg)	81.25	15.84
Waist circumference (cm)	103.04	13.53
Vigorous Physical Activity (Min/week)	70.82	468.55
Moderate Physical Activity (Min/week)	516.07	1,282.00
Walking time (Min/week)	1953.99	1,125.87
Total Physical Activity (Min/week)	2,417.72	1626.04

### 2.2 Study design and measures

#### 2.2.1 Procedure

The SE/SS‐ASMD scale was administered by several qualified therapists. On the same day, the responses of the SE and SS scale were collected, body mass index (BMI), waist circumference and a scale to assess current physical activity (International Physical Activity Questionnaire, IPAQ‐Short Version) were also measured.

#### 2.2.2 Ethics

After receiving project information verbally and in writing, study participants signed informed consent forms. Ethical approval of the study was granted by the Committee of the Faculty of Health Sciences at the University of Málaga (29/01/2015). The study was carried out in accordance with the Ethical Principles for Medical Research Practice involving Human Subjects (Declaration of Helsinki 2008). Personal data of the participants were protected in accordance with the Organized Protection of Personal Data Law 19/55.

#### 2.2.3 Measures

**The SE and SS for physical activity in people with SMDs (SE/SS‐ASMD) scale**: This scale was developed to measure the effect of SE and SS on physical activity performed by people with SMDs. To assess construct validity of the scales with regard to SE and SS, a confirmatory factor analysis (CFA) was carried out. The results of the 100 participants showed a good fit for each of the scales, supporting their single‐factor structure. Detailed study methods were as described in Cuesta‐Vargas et al. (Cuesta‐Vargas, Paz‐Lourido, Lee, & Peterson‐Besse, 2013). This modified set of SE/SS‐ASMD scales, composed of 23 questions, was employed in this study. The SE scale contained six items, with response options of “no,” “maybe,” and “yes.” Together, the three SS scales included 17 items, six family items, six staff items, and five peer items. The SS scales had response options of “no,” “yes–sometimes,” and “yes–a lot.”

**The EuroQol‐5 Dimension (EQ‐5D) questionnaire** was a brief, multi‐attribute, generic, health status measure composed of five questions with Likert response options (descriptive system), and a visual analog scale (EQ‐VAS). The latter asked patients to rate their health from 0 to 100 (the worst and best imaginable health, respectively). The descriptive system covered five dimensions of health (mobility, self‐care, everyday activities, pain and discomfort, and anxiety and depression) with five levels in each dimension (no problems, slight problems, moderate problems, severe problems, and unable to perform or extreme problems) (EuroQol Group, 1990).

International Physical Activity Questionnaire was a 27‐item self‐reporting measure of physical activity for use in adults. The scale classified light, moderate and vigorous activity, as well as sedentary behavior, over the previous seven days. Participants had to report how many days per week they had performed physical activity, and the duration in hours and minutes (Craig et  al., 2003).

### 2.3 Statistical analysis

Descriptive analysis was applied to calculate means (±standard deviation, *SD*) and medians (±interquartile range) of demographic variables and the SE/SS‐ASMD scales. This summarized the variables measured and determined the level of SE and SS for the participants. The Kaiser‐Meyer‐Olkin (KMO) test measured sampling adequacy and Bartlett's test of sphericity was used to check for redundancy between variables; KMO >0.5 was considered good, together with significant values for Bartlett's test of sphericity («IBM Kaiser‐Meyer‐Olkin measure for identity correlation matrix ”United States», 2011). Exploratory factor analysis (EFA) with maximum likelihood extraction and varimax rotation was estimated for the internal structure of the new questionnaire. To determine the structural validity of the scales, CFA was also employed. Although CFA was performed separately on each SS scale in the Cuesta‐Vargas et al. study (30), it was done on the items combined into one scale in this current study. For CFA, factor loadings of the variables were calculated and maximum likelihood estimation was employed to estimate model fit. The model fit indices included chi‐square (χ^2^), the root mean square error of approximation (RMSEA), and the comparative fit index (CFI). For RMSEA, values ≤0.08 reflected a close and reasonable fit, whereas values <0.05 indicated an excellent fit (Schumacker & Lomax, 2004). The CFI varied along a continuum of 0 to 1 in which values ≥0.80 were considered to be a satisfactory fit, and ≥0.95 reflected an excellent fit (Browne & Cudeck, 1992). Only those items whose factor loadings in the EFA were >0.40, a well‐known cutoff point of acceptability (Tabachnick & Fidell, 2001), were retained for subsequent analyses. Items with significant cross‐loadings were deleted.

To determine the internal consistency of the scale, Cronbach's α coefficients were calculated for each subscale.

The convergent criterion validation referred to the evaluation of instruments that assessed variables that measured similar constructs, or that should show a similar relationship. The relationship of these score with the variables was used to validate the SE/SS‐ASMD scale. The Pearson correlation coefficient was used to evaluate convergent validity between the SE/SS‐ASMD scale and the EQ‐5D questionnaire, and IPAQ.

All statistical analyses were conducted using the Statistical Package for Social Science for Windows version 21.0 and SPSS AMOS.

## 3 RESULTS

### 3.1 Descriptive statistics

The participants were 76% men (76 men and 24 women), and performed an average of 70.82 min per week of vigorous physical activity. Their mean BMI was 29.13, and mean waist circumference was 103.04 cm. The mean and *SD* for each of these items are shown in Table 1.

### 3.2 Exploratory factor analysis (EFA)

**Figure 1:**
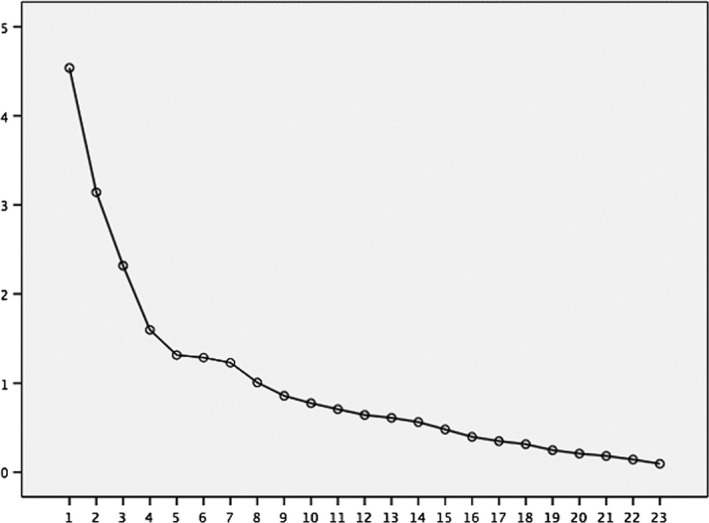
Scree plot of rotated factors

The correlation matrix, preliminarily evaluated by Bartlett's sphericity test rejected the null hypothesis of an identity matrix (degrees of freedom, *df* 253; *p* < .001), with a KMO sample adequacy measure of 0.78, indicating that the sample was adequate (Pereira, 1999). An eigenvalue >1, a value >10% of the variance and a scree test (Figure 1) were used as criteria for the extraction of factors (Cattell, 1966; Kaiser, 1960). Based on these conditions, four factors were extracted, fulfilling two of the three criteria (autovalue >1 and curve of the scree test). The total variance explained with the four factors was 70.41%.

The loading of the factors in the functional rotated structure, with a cutoff point >0.3, identified the following items in each factor (see Table 2): factor 1 (five items), 1, 2, 3, 4, and 5; factor 2 (three items), 6, 7, 8, and 9; factor 3 (three items), 10, 11, and 12; and factor 4 (four items): 13, 14, 15, and 16.

**Table 2:** Factor loading matrix after varimax rotation

	Factor			
	1	2	3	4
SE‐SMD_1	0.42			
SE‐SMD_2	0.70			
SE‐SMD_3	0.74			
SE‐SMD_4	0.59			
SE‐SMD_5	0.63			
SS‐SMD_Family_1		0.69		
SS‐SMD_Family_2		0.50		
SS‐SMD_Family_3		0.63		
SS‐SMD_Family_4		0.41		
SS‐SMD_Staff_1			0.77	
SS‐SMD_Staff_2			0.69	
SS‐SMD_Staff_3			0.76	
SS‐SMD_Peers_1				0.54
SS‐SMD_Peers_2				0.96
SS‐SMD_Peers_3				0.61
SS‐SMD_Peers_4				0.75

The varimax rotation method was used because it guarantees that we did not exclude the possibility of expressing a certain element in a factor, or in more than one factor.

### 3.3 Confirmatory factor analysis (CFA)

Most of the fit indexes were satisfactory, with CFI = 0.95 and RMSEA = 0.05 for the entire SE/SS‐ASMD scale. The Chi‐square test (χ^2^) for the four factors was significant (χ^2^ = 116.10, *df* = 98, *p* < .01).

### 3.4 Reliability

**Figure 2:**
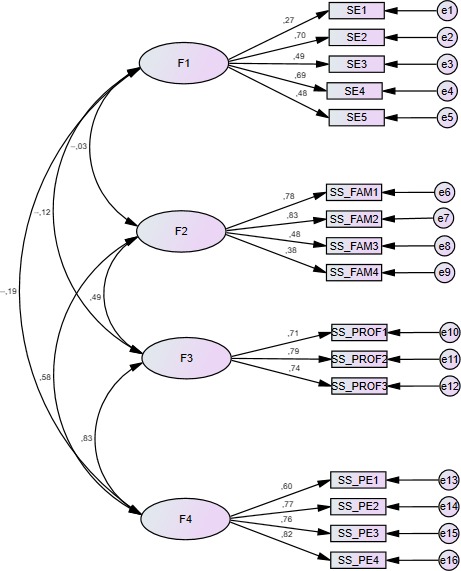
Patch way (four factors)

The Internal consistency, Cronbach's α, for the SE‐ASMD, SS‐ASMD staff, SS‐ASMD peers, and SS‐ASMD family scales were 0.76, 0.76, 0.80, and 0.80, respectively. With these results, the scale of SE and SS for physical activity seemed to be well adapted to a four‐factor model for people with SMDs (Figure 2).

### 3.5 Convergent validity criteria

Due to the lack of a gold standard for the evaluation of SE and SS in the practice of physical activity in people with SMDs, there is no tool in the current literature that evaluates these variables in this population, the scale in the current study was compared with the IPAQ (Craig et al., 2003) and with the EQ‐5D questionnaire (EuroQol Group, 1990).

Table 3 shows the correlations of the SE/SS‐ASMD scale with the IPAQ and the EQ‐5D questionnaire. Although the levels of correlation found were low (≈0.3), they were significant between the SE/SS‐ATMS scale in each of its four factors and the scales of quality of life and physical activity, with a magnitude of 0.28 (SE‐Qol) to 0.39 (Peers Support‐Total PA).

**Table 3:** Pearson correlation between SE/SS‐ASMD and EQ‐5D and IPAQ for physical activity

	Self‐efficacy	Family Support	Professional Staff	Peer Support
Quality of life	0.28*	−0.06	0.06	−0.01
Vigorous PA	0.29*	0.34**	0.28*	0.29*
Moderate PA	0.08	0.36**	0.03	0.13
Light PA	0.22	0.19	0.04	0.33*
Total PA	0.36**	0.39**	0.13	0.39**

Abbreviation: PA: physical activity.

* *p* < .05

** *p* < .01

## 4 DISCUSSION

The need to increase the levels of physical activity in people with SMDs has been studied in different places, due to its influence on health in general (Vancampfort, Stubbs, Sienaert, et al., 2015; Vancampfort, Stubbs, Ward, Teasdale, & Rosenbaum, 2015). Unfortunately, as for the rest of the world, obesity levels in Spain have progressively increased in recent decades (Sánchez‐Cruz, Jiménez‐Moleón, Fernández‐Quesada, & Sánchez, 2013). Concerns about levels of obesity are even more relevant in people with SMDs, as it greatly reduces autonomy and increases the dependence on others for activities of daily living, in addition to the large number of negative effects that it has on physical health (Clerici et al., 2014; Kwan et al., 2014; Opel et al., 2015).

It is vital to use valid and reliable measures that are culturally and linguistically adapted for the target population. The analysis of the psychometric variables of the SE/SS‐ASMD scale allowed us to obtain a new tool for researchers in the area of physical activity for people with SMDs, with a great importance attached to SE and SS promoting the inclusion of these people in physical activity programs (Davy Vancampfort, Stubbs, Sienaert, et al., 2015; Vancampfort, Stubbs, Venigalla, et al., 2015).

The adjustment values relating to the SE and SS scales were satisfactory. The determination of a four‐dimensional structure using maximum likelihood extraction was consistent with the scale validated in intellectual disability (Cuesta‐Vargas et al., 2013). However, some items were loaded in more than one factor, though this might be due to the similarity in the identification of the groups that provided support for the practice of physical activity. Thus, the scale was composed of a dimension about SE for the practice of physical activity and three dimensions related to SS (family, professional and peer).

The internal consistency values for these four dimensions, evaluated with the Cronbach's α statistic, were acceptable, being in all cases >0.7 for each of the factors of which the SE/SS‐ASMD scale was composed (Jisu, Delorme, & Reid, 2006).

Being a novel scale in the field of SMDs, a gold standard was not available for validation of convergent criteria. Instead, comparisons were made with other scales of quality of life and physical activity, showing significant correlations in at least one of the variables in each of the four factors. SE correlated significantly with both quality of life and physical activity, and SS, from relatives, professionals, and colleagues, also showed significant correlations with the physical activity performed. This confirmed that these constructs showed a relation to each other consistent with scales of SE and support for the promotion of physical activity.

### 4.1 Limitations

The present study had a series of limitations. The participants in the study population were those with SMDs, so the sample was very heterogeneous, with many psychiatric disorders. The cognitive functions of the participants were not evaluated, so this could have led to bias in their responses to the scale. In addition, there was no gold standard for validation of convergent criteria of the SE/SS‐ASMD scale. The lack of longitudinal data also did not allow the assessment of important psychometric properties, such as test‐retest reliability or sensitivity to change. The indices were satisfactory for the sample size of 100 participants; however, due to the small sample, it was not possible to divide the sample into two subsamples for separate exploratory factor analysis and confirmatory factor analysis separately.

## 5 CONCLUSION

SE and SS are key factors in achieving improvements in physical activity performed by people with SMDs. The Spanish version of the SE/SS‐ASMD scale was a useful tool, being developed to assess the influence of SE and SS on participation in physical activity (See supplementary Material 1). The promising psychometric properties of this scale shown in this study of a Spanish population indicated that it could be used in future research studies or for future interventions in people with SMDs.

## CONFLICT OF INTERESTS

None declared.

## Data Availability

The data that support the findings of this study are available on request from the corresponding author. The data are not publicly available due to privacy or ethical restrictions.

## References

[brb31510-bib-0001] Ariyabuddhiphongs, V. , & Chanchalermporn, N. (2007). A test of social cognitive theory reciprocal and sequential effects: Hope, superstitious belief and environmental factors among lottery gamblers in Thailand. Journal of Gambling Studies / Co‐Sponsored by the National Council on Problem Gambling and Institute for the Study of Gambling and Commercial Gaming, 23(2), 201–214. 10.1007/s10899-006-9035-3 17149670

[brb31510-bib-0002] Barz, M. , Lange, D. , Parschau, L. , Lonsdale, C. , Knoll, N. , & Schwarzer, R. (2015). Self‐efficacy, planning, and preparatory behaviours as joint predictors of physical activity: A conditional process analysis. Psychology & Health, 31(1), 65–78, 10.1080/08870446.2015.1070157 26155825

[brb31510-bib-0003] Bergström, G. , Börjesson, M. , & Schmidt, C. (2015). Self‐efficacy regarding physical activity is superior to self‐assessed activity level, in long‐term prediction of cardiovascular events in middle‐aged men. BMC Public Health, 15, 820 10.1186/s12889-015-2140-4 26303077 PMC4548687

[brb31510-bib-0004] Bezyak, J. L. , Berven, N. L. , & Chan, F. (2011). Stages of change and physical activity among individuals with severe mental illness. Rehabilitation Psychology, 56(3), 182–190. 10.1037/a0024207 21707200

[brb31510-bib-0005] Browne, M. W. , & Cudeck, R. (1992). Alternative ways of assessing model fit. Sociological Methods & Research, 21(2), 230–258. 10.1177/0049124192021002005

[brb31510-bib-0006] Carpiniello, B. , Primavera, D. , Pilu, A. , Vaccargiu, N. , & Pinna, F. (2013). Physical activity and mental disorders: A case‐control study on attitudes, preferences and perceived barriers in Italy. Journal of Mental Health (Abingdon, England), 22(6), 492–500. 10.3109/09638237.2013.815330 24206453

[brb31510-bib-0007] Cattell, R. B. (1966). The scree test for the number of factors. Multivariate Behavioral Research, 1, 629–637. Recuperado de Scopus. 10.1207/s15327906mbr0102_10 26828106

[brb31510-bib-0008] Citrome, L. , & Yeomans, D. (2005). Do guidelines for severe mental illness promote physical health and well‐being? Journal of Psychopharmacology (Oxford, England), 19(6 Suppl), 102–109. 10.1177/0269881105059505 16280343

[brb31510-bib-0009] Clark, M. M. , Bradley, K. L. , Jenkins, S. M. , Mettler, E. A. , Larson, B. G. , Preston, H. R. , … Douglas, K. S. V. (2015). Improvements in health behaviors, eating self‐efficacy, and goal‐setting skills following participation in wellness coaching. American Journal of Health Promotion: AJHP., 10.4278/ajhp.140627-QUAL-304 26305609

[brb31510-bib-0010] Clerici, M. , Bartoli, F. , Carretta, D. , Crocamo, C. , Bebbington, P. , & Carrà, G. (2014). Cardiovascular risk factors among people with severe mental illness in Italy: A cross‐sectional comparative study. General Hospital Psychiatry, 36(6), 698–702. 10.1016/j.genhosppsych.2014.08.005 25217493

[brb31510-bib-0011] Craig, C. L. , Marshall, A. L. , Sjöström, M. , Bauman, A. E. , Booth, M. L. , Ainsworth, B. E. , … Oja, P. (2003). International physical activity questionnaire: 12‐country reliability and validity. Medicine and Science in Sports and Exercise, 35(8), 1381–1395. 10.1249/01.MSS.0000078924.61453.FB 12900694

[brb31510-bib-0012] Cuesta‐Vargas, A. I. , Paz‐Lourido, B. , Lee, M. , & Peterson‐Besse, J. J. (2013). Adaptation and psychometric properties of the self‐efficacy/social support for activity for persons with intellectual disability scale (SE/SS‐AID) in a Spanish sample. Journal of Intellectual & Developmental Disability, 38(2), 172–176. 10.3109/13668250.2013.784959 23593928

[brb31510-bib-0013] Daumit, G. L. , Goldberg, R. W. , Anthony, C. , Dickerson, F. , Brown, C. H. , Kreyenbuhl, J. , … Dixon, L. B. (2005). Physical activity patterns in adults with severe mental illness. The Journal of Nervous and Mental Disease, 193(10), 641–646. 10.1097/01.nmd.0000180737.85895.60 16208158

[brb31510-bib-0014] Draper, C. E. , Grobler, L. , Micklesfield, L. K. , & Norris, S. A. (2015). Impact of social norms and social support on diet, physical activity and sedentary behaviour of adolescents: A scoping review. Child: Care, Health and Development, 41(5), 654–667. 10.1111/cch.12241 25809525

[brb31510-bib-0015] EuroQol Group (1990). EuroQolâ ”A new facility for the measurement of health‐related quality of life. Health Policy (Amsterdam, Netherlands), 16(3), 199–208. 10.1016/0168-8510(90)90421-9 10109801

[brb31510-bib-0016] Gao, Z. (2012). Urban Latino school children’s physical activity correlates and daily physical activity participation: A social cognitive approach. Psychology, Health & Medicine, 17(5), 542–550. 10.1080/13548506.2011.647699 22304333

[brb31510-bib-0017] Gray, K. M. , Piccinin, A. , Keating, C. M. , Taffe, J. , Parmenter, T. R. , Hofer, S. , … Tonge, B. J. (2014). Outcomes in young adulthood: Are we achieving community participation and inclusion? Journal of Intellectual Disability Research, 58(8), 734–745. 10.1111/jir.12069 23865802

[brb31510-bib-0018] Helmrich, S. P. , Ragland, D. R. , Leung, R. W. , & Paffenbarger, R. S. (1991). Physical activity and reduced occurrence of non‐insulin‐dependent diabetes mellitus. The New England Journal of Medicine, 325(3), 147–152. 10.1056/NEJM199107183250302 2052059

[brb31510-bib-0019] Hoffmann, K. D. , Walnoha, A. , Sloan, J. , Buddadhumaruk, P. , Huang, H.‐H. , Borrebach, J. , … Burke, J. G. (2015). Developing a community‐based tailored exercise program for people with severe and persistent mental illness. Progress in Community Health Partnerships: Research, Education, and Action, 9(2), 213–227. 10.1353/cpr.2015.0045 PMC4883579 26412763

[brb31510-bib-0020] Huffman, K. M. , Pieper, C. F. , Hall, K. S. , St Clair, E. W. , & Kraus, W. E. (2015). Self‐efficacy for exercise, more than disease‐related factors, is associated with objectively assessed exercise time and sedentary behaviour in rheumatoid arthritis. Scandinavian Journal of Rheumatology, 44(2), 106–110. 10.3109/03009742.2014.931456 25222824 PMC4356639

[brb31510-bib-0022] Jisu, H. , Delorme, D. E. , & Reid, L. N. (2006). Perceived third‐person effects and consumer attitudes on prevetting and banning DTC advertising. Journal of Consumer Affairs, 40(1), 90–116. 10.1111/j.1745-6606.2006.00047.x

[brb31510-bib-0023] Kaiser, H. F. (1960). The application of electronic computers to factor analysis. Educational and Psychological Measurement, 20, 141–151. 10.1177/001316446002000116

[brb31510-bib-0024] Kwan, C. L. , Gelberg, H. A. L. , Rosen, J. A. , Chamberlin, V. , Shah, C. , Nguyen, C. , … Ames, D. (2014). Nutritional counseling for adults with severe mental illness: Key lessons learned. Journal of the Academy of Nutrition and Dietetics, 114(3), 369–374. 10.1016/j.jand.2013.12.005 24534370

[brb31510-bib-0025] Lee, M. , Peterson, J. J. , & Dixon, A. (2010). Rasch calibration of physical activity self‐efficacy and social support scale for persons with intellectual disabilities. Research in Developmental Disabilities, 31(4), 903–913. 10.1016/j.ridd.2010.02.010 20363109

[brb31510-bib-0026] Leon, A. S. , Connett, J. , Jacobs, D. R. , & Rauramaa, R. (1987). Leisure‐time physical activity levels and risk of coronary heart disease and death. The Multiple Risk Factor Intervention Trial. JAMA, 258(17), 2388–2395. 10.1001/jama.1987.03400170074026 3669210

[brb31510-bib-0027] Mendonça, G. , & de Farias Júnior, J. C. (2015). Physical activity and social support in adolescents: Analysis of different types and sources of social support. Journal of Sports Sciences, 33(18), 1942–1951. 10.1080/02640414.2015.1020842 25751023

[brb31510-bib-0028] Muñoz, M. , Perez‐Santos, E. , Crespo, M. , & Guillen, M. I. (2009). Estigma y enfermedad mental. Complutense.

[brb31510-bib-0029] Naslund, J. A. , Aschbrenner, K. A. , Scherer, E. A. , Pratt, S. I. , Wolfe, R. S. , & Bartels, S. J. (2015). Lifestyle intervention for people with severe obesity and serious mental illness. American Journal of Preventive Medicine, 10.1016/j.amepre.2015.07.012 PMC4718763 26385164

[brb31510-bib-0030] Opel, N. , Redlich, R. , Grotegerd, D. , Dohm, K. , Heindel, W. , Kugel, H. , … Dannlowski, U. (2015). Obesity and major depression: Body‐mass index (BMI) is associated with a severe course of disease and specific neurostructural alterations. Psychoneuroendocrinology, 51, 219–226. 10.1016/j.psyneuen.2014.10.001 25462895

[brb31510-bib-0031] Osborn, D. P. J. , Nazareth, I. , & King, M. B. (2007). Physical activity, dietary habits and Coronary Heart Disease risk factor knowledge amongst people with severe mental illness: A cross sectional comparative study in primary care. Social Psychiatry and Psychiatric Epidemiology, 42(10), 787–793. 10.1007/s00127-007-0247-3 17721669

[brb31510-bib-0032] Paffenbarger, R. S. , Hyde, R. T. , Wing, A. L. , & Hsieh, C. C. (1986). Physical activity, all‐cause mortality, and longevity of college alumni. The New England Journal of Medicine, 314(10), 605–613. 10.1056/NEJM198603063141003 3945246

[brb31510-bib-0033] Pereira, J. C. R. (1999). Análise de Dados Qualitativos: Estratégias Metodológicas para as Ciências da Saúde Humanas e Sociais. EdUSP.

[brb31510-bib-0034] Perez‐Cruzado, D. , Cuesta‐Vargas, A. I. , Vera‐Garcia, E. , & Mayoral‐Cleries, F. (2018). The relationship between quality of life and physical fitness in people with severe mental illness. Health and Quality of Life Outcomes, 16(1), 82 10.1186/s12955-018-0909-8 29720196 PMC5930752

[brb31510-bib-0035] Plotnikoff, R. C. , Gebel, K. , & Lubans, D. R. (2014). Self‐efficacy, physical activity, and sedentary behavior in adolescent girls: Testing mediating effects of the perceived school and home environment. Journal of Physical Activity & Health, 11(8), 1579–1586. 10.1123/jpah.2012-0414 24733181

[brb31510-bib-0036] Sánchez‐Cruz, J.‐J. , Jiménez‐Moleón, J. J. , Fernández‐Quesada, F. , & Sánchez, M. J. (2013). Prevalence of child and youth obesity in Spain in 2012. Revista Española De Cardiología (English Ed.), 66(5), 371–376. 10.1016/j.rec.2012.10.012 24775819

[brb31510-bib-0037] Schumacker, R. E. , & Lomax, R. G. (2004). A beginner’s guide to structural equation modeling. Lawrence Erlbaum Associates.

[brb31510-bib-0038] Shor, R. , & Shalev, A. (2014). Barriers to involvement in physical activities of persons with mental illness. Health Promotion International,31(1):116–23. 10.1093/heapro/dau078 25204451

[brb31510-bib-0039] Soundy, A. , Freeman, P. , Stubbs, B. , Probst, M. , & Vancampfort, D. (2014). The value of social support to encourage people with schizophrenia to engage in physical activity: An international insight from specialist mental health physiotherapists. Journal of Mental Health (Abingdon, England), 23(5), 256–260. 10.3109/09638237.2014.951481 25222369

[brb31510-bib-0040] Tabachnick, B. G. , & Fidell, L. S. (2001). Using multivariate statistics. Needham, MA: Allyn and Bacon.

[brb31510-bib-0042] Vancampfort, D. , Stubbs, B. , Sienaert, P. , Wyckaert, S. , De Hert, M. , Rosenbaum, S. , & Probst, M. (2015). What are the factors that influence physical activity participation in individuals with depression? A review of physical activity correlates from 59 studies. Psychiatria Danubina, 27(3), 210–224. 26400128

[brb31510-bib-0043] Vancampfort, D. , Stubbs, B. , Sienaert, P. , Wyckaert, S. , De Hert, M. , Soundy, A. , & Probst, M. (2016). A comparison of physical fitness in patients with bipolar disorder, schizophrenia and healthy controls. Disability and Rehabilitation, 38(20), 2047–2051. 10.3109/09638288.2015.1114037 26727888

[brb31510-bib-0044] Vancampfort, D. , Stubbs, B. , Venigalla, S. K. , & Probst, M. (2015). Adopting and maintaining physical activity behaviours in people with severe mental illness: The importance of autonomous motivation. Preventive Medicine, 81, 216–220. 10.1016/j.ypmed.2015.09.006 26386141

[brb31510-bib-0045] Vancampfort, D. , Stubbs, B. , Ward, P. B. , Teasdale, S. , & Rosenbaum, S. (2015). Integrating physical activity as medicine in the care of people with severe mental illness. The Australian and New Zealand Journal of Psychiatry, 49(8), 681–682. 10.1177/0004867415590831 26041791

[brb31510-bib-0046] Willis, E. (2015). Patients’ self‐efficacy within online health communities: Facilitating chronic disease self‐management behaviors through peer education. Health Communication, 31(3), 299–307. 10.1080/10410236.2014.950019 26325224

[brb31510-bib-0047] Zhang, N. , Campo, S. , Yang, J. , Janz, K. F. , Snetselaar, L. G. , & Eckler, P. (2015). Effects of social support about physical activity on social networking sites: applying the theory of planned behavior. Health Communication, 30(12), 1277–1285. 10.1080/10410236.2014.940669 26086237

