# Supplementary material for: Adaptation and validation of a scale of self‐efficacy and social support for physical activity in Spanish patients with severe mental disorders
Source: Brain Behav. 2019 Dec 26;10(2):e01510. doi: 10.1002/brb3.1510 (PMC7010581; doi:10.1002/brb3.1510)
Supplement: Supplementary file 1 [file BRB3-10-e01510-s001.docx]

**Annexed 1: SE/SS-ASMD scale**

| 1 | Do you think you can make time for physical activity most days? [¿Crees que puedes sacar tiempo para hacer actividades físicas casi todos los días?] |
| --- | --- |
| 2 | Do you think you can do physical activities even when you feel sad or depressed? [¿Crees que puedes hacer actividades físicas incluso cuando te sientes triste o depresivo?] |
| 3 | Do you think you can do physical activities even after a day of long and hard work? [¿Crees que puedes hacer actividades físicas incluso después de un día de trabajo largo y duro?] |
| 4 | Do you think you can do daily physical activities that you are tired or low energy? [¿Crees que puedes hacer actividades físicas los días que te encuentras cansado o con poca energía?] |
| 5 | Do you think you can do physical activity when you're lazy? [¿Crees que puedes hacer actividades físicas cuando estas perezoso?] |
| 6 | Does anyone in your family makes physical activities with you? [¿Alguien en tu familia hace actividades físicas contigo?] |
| 7 | Does anyone in your family planning physical activities when you spend time together? [¿Alguien de tu familia planifica actividades físicas cuando pasáis tiempo juntos?] |
| 8 | Does anyone in your family shows you how to do physical activity? [¿Alguien de tu familia te muestra cómo hacer actividades físicas?] |
| 9 | Does anyone in your family takes you somewhere so you do physical activity when you need him? [¿Alguien de tu familia te lleva a algún sitio para que hagas actividades físicas cuando lo necesitas?] |
| 10 | Do professionals who work with you plan to do physical activity when you spend time together? [¿Los profesionales que trabajan contigo planean hacer actividades físicas cuando pasáis tiempo juntos?] |
| 11 | Are you working professionals teach you how to do physical activity? [¿Los profesionales que trabajan contigo te enseñan cómo hacer actividades físicas?] |
| 12 | Are the professionals working with you say you’re good at physical activities? [¿Los profesionales que trabajan contigo te dicen que eres bueno en las actividades físicas?] |
| 13 | Have any of your peers (or friends) remind you do physical activity? [¿Alguno de tus compañeros/as de piso (o amigos/as) te recuerdan hacer actividades físicas?] |
| 14 | Have any of your peers (or friends) do physical activities with you? [¿Alguno de tus compañeros/as de piso (o amigos/as) hacen actividades físicas contigo?] |
| 15 | Have any of your peers (or friends) asked you if you do physical activities with them, or have thought? [¿Alguno de tus compañeros/as de piso (o amigos/as) te preguntan si haces actividades físicas con ellos, o lo han pensado?] |
| 16 | Have any of your peers (or friends) say you are good at physical activities? [¿Alguno de tus compañeros/as de piso (o amigos/as) te dicen que eres bueno haciendo actividades físicas?] |
